# Sarcopenia and ischemic stroke outcomes after endovascular revascularization: results of a retrospective, cohort study

**DOI:** 10.3389/fneur.2026.1732174

**Published:** 2026-02-10

**Authors:** Lea Maria Bumann, Bijan Zendeh Zartoshti, Ulrike Voßmann, Daniel Cantré, Artem Rafaelian, Daniel Dubinski, Alexander Storch, Matthias Wittstock

**Affiliations:** 1 Department of Neurology, University Medical Center Rostock, Rostock, Germany; 2 Institute of Diagnostic and Interventional Radiology, Pediatric Radiology and Neuroradiology, University Medical Center Rostock, Rostock, Germany; 3 Department of Neurosurgery, University Medical Center Rostock, Rostock, Germany

**Keywords:** endovascular therapy, outcome, prognosis, sarcopenia, stroke

## Abstract

Stroke is a major cause of disability and mortality, with its incidence increasing with age. Despite advances in acute stroke treatment, functional outcomes in elderly patients are not always as favorable as expected. Therefore, additional efforts are required to identify reliable prognostic markers and improve patient outcomes. Sarcopenia has been recognized as a negative factor influencing functional outcomes after ischemic stroke. Temporalis muscle thickness (TMT), assessed on routine imaging, has emerged as a potential surrogate marker for sarcopenia; however, its prognostic value in stroke patients has not yet been conclusively established. This study aimed to evaluate the association between TMT and functional outcome, assessed using the modified Rankin Scale (mRS) at hospital discharge, and in-hospital death. In this retrospective cohort study, we included 152 consecutive patients (median age, 74 years [IQR, 63–83]; 53% male) admitted to the Department of Neurology at the University of Medicine Rostock who underwent endovascular revascularization (EVT) for acute ischemic stroke. TMT was determined from initial cranial CT scans. Due to the small sample size, we used a uniform TMT cutoff value (5.78 mm) for sarcopenia. The primary endpoint was the mRS at discharge. Sarcopenic patients were older (*p* < 0.001), more often female (*p* < 0.001), and had a lower BMI (*p* = 0.045). They also had significantly worse functional outcomes (*p* = 0.006) and higher comorbidity burdens (atrial fibrillation, *p* = 0.023; arterial hypertension, *p* = 0.024; smoking, *p* = 0.020). Poor outcome was significantly associated with sarcopenia, but this association lost significance after adjustment. In the group comparison regarding mortality, deceased patients had lower TMT and were more frequently sarcopenic. However, these associations also lost significance in the multivariate model.

## Introduction

Stroke is a major cause of disability and mortality worldwide (1). Its incidence increases with age (2). Despite improvements in care and treatment, the functional outcome in elderly stroke patients is not always as favorable as expected. Sarcopenia has been discussed as a contributing factor in this context (3, 4). Sarcopenia—defined as the loss of skeletal muscle mass—has been shown to increase after stroke. Both sarcopenia and stroke have been associated with worse functional outcomes (3, 4). This highlights the importance of measuring skeletal muscle mass as a measure of sarcopenia in older stroke patients with respect to functional outcomes.

Diagnostic tools for assessing sarcopenia rely on methods that estimate muscle quantity, including magnetic resonance imaging (MRI) and computed tomography (CT) scans of the lumbar muscles obtained during abdominal scans (5–7). More recently, estimation of temporalis muscle thickness (TMT) has become a favorable approach for sarcopenia measurement, as it can be easily, quickly, and reliably performed during routine imaging. This has been demonstrated in cerebellar ischemia and traumatic brain injury (8, 9). Evidence of the prognostic value of TMT in EVT for ischemic stroke is limited, and the relevance of TMT measurements is not fully clarified (10, 11). The current study aimed to investigate the association between TMT, as a measure of sarcopenia, and functional outcomes and to assess mortality in elderly patients undergoing EVT for acute ischemic stroke at hospital discharge.

## Methods

### Study design

**Figure 1:**
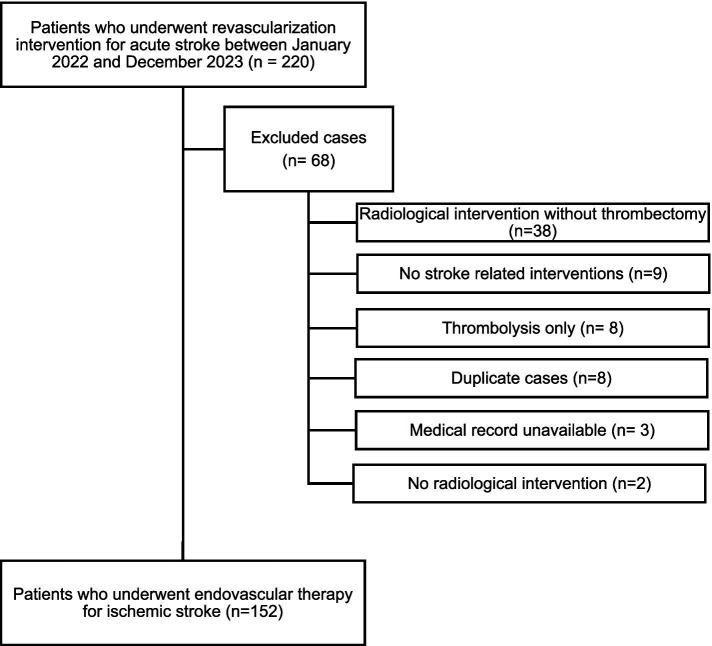
Study flowchart.

In this retrospective cross-sectional cohort study, we screened the hospital charts of 220 consecutive patients admitted to the Department of Neurology of the University Medicine Rostock between January 2022 and December 2023 undergoing revascularization procedures (thrombolysis, endovascular thrombectomy, or both) for acute ischemic stroke. After exclusion of patients with insufficient clinical data due to wrong coding or duplicate records, a total of 152 patients were included in the final analysis (see Figure 1 for study flowchart). This study was conducted in accordance with the amended Declaration of Helsinki and was approved by the local ethics committee (A 2024–0039).

### Patients

All patients received standard-of-care treatment according to the European Stroke Organization Guidelines for ischemic stroke (12). Basic characteristics such as age, sex, body mass index (BMI), pre-morbid disability measured by the individual’s category in the Clinical Frailty Scale (CFS) from the Canadian Study of Health and Aging (CSHA) (13), cardiovascular risk factors, presence of advance directive, and imaging data, as well as therapeutic procedures of the acute phase and length of hospitalization were obtained. Clinical severity of stroke was assessed using the National Institutes of Health Stroke Scale (NIHSS) and the ASPECTS score (14).

The topology of stroke was assessed by an experienced board-certified neuroradiologist (D.C.) who was blinded to the hypothesis investigated in this study using cerebral CT or MRI. The occurrence of large vessel occlusion (LVO) was determined. Stroke volumes were measured on CT or MRI scans using Brainlab software (Brainlab AG), and the ASPECTS score was determined (14). After EVT, flow restoration at the end of each procedure was graded using the modified Treatment In Cerebral Infarction (mTICI) scale, with optimal recanalization corresponding to a score of 2b-3 (15).

The mRS score at discharge from the hospital represents the primary outcome measure. An mRS score between 0 and 3 was considered a good functional outcome, scores between 4 and 6 represented a bad outcome, and a score of 6 represented in-hospital death, which was assessed separately (16). A 24-h follow-up CT scan was assessed to identify complications such as brain bleeding events (secondary intracranial hemorrhage ICH), defined according to the European Cooperative Acute Stroke Study (ECASS II) classification (17).

### TMT measurement

TMT was assessed on CT or MRI scans at admission according to the method presented previously by Ravera et al. and Steindl et al. (18, 19). In detail, TMT was manually measured on the patient’s baseline brain CT scan using the method introduced by Katsuki et al. (20). Slice thickness was set at 5 mm, and the CT axial image was manually adjusted to obtain a symmetric cross-section. TMT was measured bilaterally, perpendicular to the long axis of the temporal muscle. Three determinations were taken for each side: one at the level of the orbital roof, identified by comparing a sagittal view, another 5 mm above the orbital roof, and the last at 5 mm below the orbital roof. The arithmetic mean of the three measurements was calculated for both the left and right sides. Once the right and left means were obtained, the final TMT, expressed in millimeters, was measured by calculating the arithmetic mean between the two values. Based on the mean TMT values, patients were further divided into two groups: sarcopenic (TMT < 5.78 mm) and non-sarcopenic (TMT ≥ 5.78 mm). Because of the small sample size, female and male patients were analyzed together in each group. An analysis of sex-specific differences according to sex-specific TMT cutoffs was performed according to Steindl et al. The results are provided in Supplementary Tables S1, S2.

### Statistical analysis

For statistical comparisons between groups, the Mann–Whitney *U*-test or Kruskal–Wallis test was used for comparison of parametric data, and the Pearson’s chi-squared test or Fisher’s exact test for comparison of non-parametric data, as appropriate. For ordinal data, the Jonckheere–Terpstra test was applied. To test whether there was an association between categorical clinical variables and the outcome of interest, univariate and multivariate binary logistic (for in-hospital death as the dependent outcome variable) or ordinal regression analyses (for mRS as the dependent outcome measure) were performed. To select relevant covariates, we performed the Mann–Whitney *U*-test and the chi-squared/Fisher’s exact test in combination with univariate regression models to determine the predictive values and odds ratios (ORs) with 95% confidence intervals (95% CIs) of the candidate covariates age, sex, NIHSS at admission, stroke volume, intracerebral hemorrhage, length of hospital stay, aspiration pneumonia, and atrial fibrillation (for detailed information, see Supplementary Tables S3, S4). Before calculating multivariate regressions, assumptions of normality, homoscedasticity, independence of errors, and absence of multicollinearity were checked. The results (variance inflation factor values) are shown in the Supplementary Tables S5, S6.

Analyses were conducted using R software, version 4.4.1 (R Foundation for Statistical Computing, Vienna, Austria) via RStudio version 2024.09.0 (Posit Software, PBC), and all *p*-values were two-sided, and values of P less than 0.05 were considered statistically significant. Due to the limited sample size in our retrospective study, adjustment of *p-*values was not carried out to preserve statistical power.

## Results

### Demographic and clinical characteristics

A total of 220 consecutive patients treated by EVT for LVO in ischemic stroke were screened; 68 were excluded due to wrong coding or unavailable clinical data (Figure 1). At admission, all patients except one received CT imaging (1 patient underwent MR imaging, 0.7%). A total of 152 ischemic stroke patients with LVO were included (median age 74 years [IQR 63–83 years]; 53.0% men). The median value of NIHSS at admission was 16 (IQR 10–19). Detailed demographic and clinical characteristics are illustrated in Table 1.

**Table 1:** Demographics, baseline clinical characteristics and management data of study cohort.

Baseline characteristics	Total cohort (*n* = 152)	Non-sarcopenic group (*n* = 74)	Sarcopenic group (*n* = 78)	*p* value
Males/Females, *n* (%)	80 (52.6%)/72 (47.4%)	54 (72.9%)/20 (27.0%)	26 (33.3%)/52 (66.6%)	**<0.001** ^**$**^
Age (years), Median (IQR)	74.0 (63.0–83.0)	68.0 (58.2–77.7)	82.0 (69.2–85.7)	**<0.001** ^*^
BMI (kg/m^2^), Median (IQR)	26.1 (23.3–29.7)	26.3 (24.3–29.9)	25.2 (22.1–28.3)	**0.045** ^*^
TMT (mm), mean ± SD	5.78 ± 1.78	7.29 ± 1.03	4.35 ± 0.98	<0.001*
Sarcopenia, *n* (%)	78 (51.3%)	-	-	**-**
CFS at admission, Median (IQR)	2 (2–3)	2 (2)	2 (2–4)	0.038*
Length of hospital stay (days), Median (IQR)	11 (6–15)	10.5 (7–15.75)	11 (6–15)	0.921^*^
GCS at admission, Median (IQR)	11 (8–15)	12 (8–15)	11 (8.25–15)	0.956^*^
NIHSS at admission, Median (IQR)	16 (10–19)	15 (9–22)	16 (10–19)	0.924^*^
Stroke-related parameters				
ASPECTS score, Median (IQR)	8 (6–9)	8 (7–9)	8 (6–9)	0.247*
Stroke volume (ml), Median (IQR)	22.2 (4.4–76.3)	20.5 (4.8–70.65)	23.3 (3.81–78.8)	0.857^*^
Thrombectomy, *n* (%)	56 (36.8%)	30 (40.5%)	26 (33.3%)	0.357^$^
Systemic thrombolysis+thrombectomy, *n* (%)	96 (63.2%)	44 (59.4%)	52 (66.6%)	0.357^$^
mTICI 2b-3, *n* (%)	127 (83.6%)	60 (81.1%)	67 (85.8%)	0.303^$^
Complications				
Aspiration Pneumonia, *n* (%)	43 (28.3%)	20 (27.0%)	23 (29.4%)	0.736^$^
Intracerebral hemorrhage, *n* (%)	23 (15.1%)	10 (13.5%)	13 (16.6%)	0.588^$^
Comorbidities				
Arterial hypertension, *n* (%)	115 (75.7%)	50 (67.6%)	65 (83.3%)	**0.024** ^**$**^
Diabetes mellitus, *n* (%)	39 (25.7%)	16 (21.6%)	23 (29.5%)	0.267^$^
Hyperlipoproteinemia, *n* (%)	64 (42.1%)	32 (43.2%)	32 (41.0%)	0.834^$^
Atrial fibrillation, *n* (%)	74 (48.7%)	29 (39.2%)	45 (57.7%)	**0.023** ^**$**^
Alcohol abuse, *n* (%)	12 (7.8%)	9 (12.2%)	3 (3.8%)	0.071^$^
Smoking, *n* (%)	33 (21.7%)	22 (29.7%)	11 (14.1%)	**0.020** ^**$**^

Values are median and interquartile ranges (IQR, in brackets), mean values ± standard deviation (SD) or *n* (%). Significant findings in bold.

BMI, Body Mass Index; CFS, Clinical Frailty Scale; GCS, Glasgow Coma Scale; NIHSS, National Institute of Health Stroke Scale; mRS, modified ranking scale; mTICI, modified treatment in cerebral infarction; TMT, temporalis muscle thickness.

*Mann-Whitney U-test.

^$^Pearson Chi^2^ or Fisher exact test as appropriate.

### Association between TMT and demographic and clinical characteristics

TMT values measured in the study cohort were significantly higher in male patients than in female patients (median [IQR] value 6.7 [5.6–7.6] vs. 4.7 [3.8–6.2]; *p* < 0.001). Significant inverse correlation (Spearman’s rho, *p*-value) could be found for age (−0.46, 95% CI: −0.57 to −0.32; *p* < 0.001) and for CFS (−0.27, 95% CI: −0.42–0.10; *p* < 0.001). Regarding the results of the sex-specific analysis of clinical data, see Supplementary Tables S1, S2.

Atrial fibrillation also showed a weak but statistically significant negative correlation with TMT (−0.18, 95% CI: −0.33, −0.02; *p* = 0.028). In contrast, alcohol abuse (0.26, 95% CI: 0.14, 0.40; *p* = 0.001) and smoking (0.24, 95% CI: 0.09, 0.39; *p* = 0.003) were both positively associated with TMT. Further analysis did not reveal any correlations with arterial hypertension, diabetes, and hyperlipoproteinemia (see Supplementary Tables S3, S4).

### Association between sarcopenia and demographic and clinical characteristics

The complete cohort was divided into a sarcopenic (51.3%) group and a non-sarcopenic group (48.7%; Table 1) (19). Sarcopenic patients were significantly older than non-sarcopenic patients (Table 1), and the frequency of sarcopenia displayed a clear age-dependency: 14% in patients <50 years, 12% in those aged 50–59 years, 24% in those aged 60–69 years, and 20% in those aged 70–79 years, and 39% in those aged 80 + years (*p* < 0.001, Spearman’s rho = −0.46). Regarding demographic and clinical characteristics, sarcopenic patients were more frequently female, had a greater pre-morbid disability, and presented a lower BMI at admission. In contrast, no significant differences were observed in NIHSS at admission, stroke volumes, and mTICI between non-sarcopenic and sarcopenic patients. The frequency of hypertension and atrial fibrillation (AF) was higher in sarcopenic patients than that in non-sarcopenic patients (Table 1). There were no differences regarding the etiology of stroke or the rate of pneumonia between sarcopenic and non-sarcopenic patients (Table 1).

### Association between TMT and functional outcome at hospital discharge

TMT was associated with major outcome parameters—mRS and death—at hospital discharge in univariate regression models, with higher TMT values linked to more favorable outcomes (see Table 1 for respective ORs). However, after adjusting for ORs using multivariate regression models that included variables influencing the outcome, the analysis of outcomes revealed no significant association between TMT and functional outcome or death at hospital discharge (Table 2).

**Table 2:** Odds ratios for functional outcomes by TMT and by non-sarcopenic vs. sarcopenic patient group.

Functional outcome	Outcome rate^a^			Univariate logistic/ordinal regression^c^		Multivariate logistic/ordinal regression^d^	
	Non-sarcopenic (*n* = 74)	Sarcopenic (*n* = 78)	*p* value^b^	Unadjusted Odds ratio (95% CI)	*p* value	Adjusted Odds ratio (95% CI)	*p* value
TMT (mm) as marker for sarcopenia							
mRS at discharge				0.81 (0.69–0.95)	**0.008**	0.79 (0.59–1.04)	0.094
mRS 6 (death) at discharge				0.74 (0.58–0.92)	**0.008**	0.70 (0.34–1.31)	0.280
Non-sarcopenic vs. sarcopenic group							
mRS at discharge, Median (IQR)	3.0 (2.0–5.0)	5.0 (3.0–6.0)	**0.006**	2.24 (1.26–3.96)	**0.006**	1.65 (0.66–4.14)	0.283
mRS 6, (death) at discharge *n* (%)	11 (15.0%)	26 (33.0%)	**0.008**	2.86 (1.32–6.56)	**0.010**	1.59 (0.22–12.03)	0.641

mRS, Modified ranking scale; TMT, temporal muscle thickness.

a Number and (%) for death rate and median (IQR) for mRS.

b *p* values are from Pearson Chi^2^ tests (death rate) or Jonckheere-Terpstra tests (mRS).

c Odds ratios and *p* values from univariate ordinal or binary logistic regression analyses. An Odds ratio >1 for the continuous variable (TMT) indicates a higher risk at higher values of TMT, while an Odds ratio >1 for dichotomous risk factor (non-sarcopenic vs. sarcopenic group) indicates a higher risk in the sarcopenic group.

d Adjustment of Odds ratios for relevant covariates were performed by multivariate ordinal or binary logistic regression with predictive variables from univariate analyses (covariates for data at hospital discharge: age, NIHSS at admission, Clinical Frailty Score at admission, stroke volume, ASPECT-Score, mTICI, hyperlipoproteinemia, aspiration pneumonia, intracerebral hemorrhage, length of hospital stay; covariates for mRS 6 at discharge: age, NIHSS at admission, Clinical Frailty Score at admission, stroke volume, ASPECT-Score, aspiration pneumonia, length of hospital stay). Significant values are displayed in bold.

### Association between sarcopenia and functional outcome at hospital discharge

**Figure 2:**
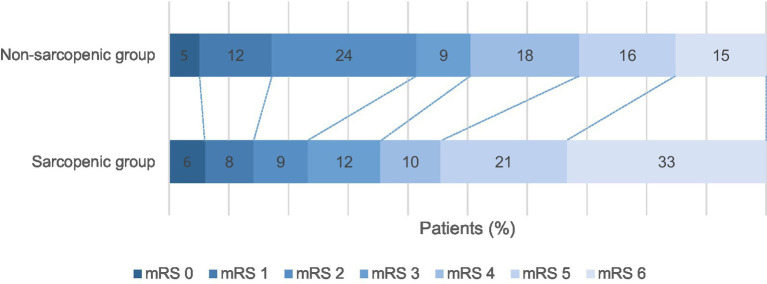
Outcomes at hospital discharge. Modified Rankin scale scores at discharge from hospital. Shown are the results of the ordinal analysis of the modified Rankin scale scores at discharge from the hospital. Scores range from 0 to 6, with 0 indicating no neurologic deficit, 1 indicating no clinically significant disability, 2 indicating slight disability (able to handle own affairs without assistance but unable to carry out all previous activities), 3 indicating moderate disability requiring some help (e.g., with shopping, cleaning, and finances but able to walk unassisted), 4 indicating moderately severe disability (unable to attend own body needs without assistance and unable to walk unassisted), 5 indicating severe disability (requiring constant nursing care and attention), and 6 indicating death. There was a significant difference between the non-sarcopenic and sarcopenic patient group at discharge (unadjusted common odds ratio: 2.24; 95% CI: 1.26–3.96; *p* = 0.006).

In Figure 2, there was a significant difference in functional outcome and in-house death between sarcopenic and non-sarcopenic patients at hospital discharge (unadjusted common OR: 2.24; 95% CI: 1.26 to 3.96; *p* = 0.006 and 2.86; 95% CI: 1.32–6.56; *p* = 0.010, respectively). After adjusting for ORs using multivariate regression models that included variables influencing the outcome, sarcopenia and functional outcome at hospital discharge (adjusted OR: 1.65; 95% CI: 0.66 to 4.14; *p* = 0.283) and fatal outcome (death) (adjusted OR: 1.59; 95% CI: 0.22 to 12.03; *p* = 0.641; Table 2) lost significant associations.

### Risk factors for functional outcome at hospital discharge

The univariate regression analysis revealed that several clinical candidate risk factors of unfavorable outcome, such as age, pneumonia, and major stroke imaging parameters, including stroke volume and secondary intracerebral hemorrhage, were associated with poor functional outcome at hospital discharge (see Supplementary Table S3). The multivariate regression analysis confirmed that the CFS at admission, stroke volume, and pneumonia were significant predictors of functional outcome at hospital discharge (Supplementary Table S4).

## Discussion

The present study aimed to investigate the relationship between sarcopenia, assessed by TMT measurement, and functional outcomes, including hospital discharge and in-hospital mortality, in patients undergoing EVT for LVO ischemic stroke.

### Association between functional outcome and sarcopenia

An analysis of functional outcomes (mRS at discharge) revealed an association between sarcopenia and adverse outcomes. However, this association was no longer significant after adjusting for candidate covariates in the multivariate ordinal regression. Regarding mortality (mRS = 6 at discharge), the univariate analysis showed a clear association with sarcopenia: crude mortality was 33% in the sarcopenic group vs. 15% in the non-sarcopenic group (*p* = 0.008). This difference was also reflected in the unadjusted logistic regression analysis, indicating that sarcopenic patients had an almost threefold increased risk of death during their hospital stay. After conducting a multivariate analysis that included important covariates such as age, stroke severity (NIHSS), frailty (CFS), infarct size, pneumonia, ASPECTS score, and others, the association lost statistical significance. There was no significant independent effect of sarcopenia on inpatient mortality. This attenuation of the effect suggests that mortality in sarcopenic patients is primarily explained by other, concurrent risk factors rather than by sarcopenia alone. In particular, age, frailty, and complications such as pneumonia or large infarctions play a dominant role. These findings are in contrast to previously published data, which demonstrated a significantly elevated 90-day mortality in sarcopenic patients (10).

### Differences between sarcopenic and non-sarcopenic patients

Significant differences were found between the sarcopenic and non-sarcopenic groups across several baseline variables. The CFS was significantly higher in the sarcopenic group. In line with previous published findings, sarcopenia was significantly more common in women, and sarcopenic patients were significantly older (21–23). As expected, both groups differed significantly in body mass index and TMT. Comorbidities such as hypertension and atrial fibrillation were more common in the sarcopenic group, which has been well documented in the literature (24). Regarding risk behavior, alcohol and tobacco consumption were more frequent in the non-sarcopenic group. This finding contrasts with the findings reported by Lin et al. (11), which showed no association between smoking and outcome in sarcopenic patients. Our findings might reflect poor general health and limited health awareness in the rural areas of Mecklenburg-Vorpommern (25, 26).

### TMT as a measure of sarcopenia

TMT was considered a continuous variable in the analysis to examine its association with functional outcome and mortality after ischemic stroke. The results revealed that higher TMT was associated with significantly better functional outcome at discharge. Specifically, for each additional millimeter of TMT, the probability of a worse mRS score decreased by approximately 22%. These findings underscore the importance of TMT as a potential biological marker of functional reserve and general health status. Patients with higher TMT scores generally appear more robust and recover better functionally after stroke. Regarding mortality (mRS = 6), a significant association was also observed in the univariate analysis, but it was not maintained in the multivariate analysis. This finding indicates that, although higher TMT is associated with a trend toward lower mortality, this effect is not independent of other relevant influencing factors such as age, comorbidities, or stroke severity.

### Limitations

The present study has certain limitations. The main limitation stems from its retrospective nature and the relatively small sample size, resulting in insufficient statistical power to detect significant differences in functional outcomes and mortality at discharge after adjustment, even if such differences actually exist. The primary outcome captures acute morbidity and mortality but fails to assess durable neurological recovery reflected in a standard 90-day endpoint. Therefore, the interpretation of TMT values is restricted to acute patient events. Another potential limitation is that the relationship between TMT and outcome could be influenced by including frailty in the multivariate model, as TMT itself is considered a surrogate of frailty. The finding that CFS (a clinical measure) was an independent predictor, while TMT (an image-based measure) was not, implies that TMT may be clinically redundant when CFS is assessed in the acute setting. However, the combined use of clinical and image-based metrics may appear redundant, as they address complementary approaches to the same problem. In the acute setting, image-based measures such as TMT offer the advantage of being rapid and independent of patient cooperation and anamnestic data.

### Clinical implications

In summary, this investigation indicates that TMT is an interesting marker of short-term risk but not an independent predictor of functional outcome and mortality after EVT for LVO in ischemic stroke patients. TMT likely reflects a patient’s general health and nutritional status, as well as their functional reserve—factors that primarily influence recovery rather than the immediate probability of survival. In-house mortality is strongly influenced by other factors, such as age, comorbidities (e.g., pneumonia), stroke severity (NIHSS), or intracranial hemorrhage. While TMT and group affiliation (sarcopenia, yes/no) correlate with poorer general health, they do not directly correlate with acute fatal complications. A multivariate adjustment eliminates the effect of sarcopenia, as many of these covariates are themselves strong predictors of death. Despite the loss of significance in the multivariate analysis, TMT may still be a useful marker of sarcopenia, and larger study cohorts may prove this assumption.

## Data Availability

The original contributions presented in the study are included in the article/Supplementary material, further inquiries can be directed to the corresponding author.
