# Supplementary material for: Sarcopenia and ischemic stroke outcomes after endovascular revascularization: results of a retrospective, cohort study
Source: Front Neurol. 2026 Feb 10;17:1732174. doi: 10.3389/fneur.2026.1732174 (PMC12929099; doi:10.3389/fneur.2026.1732174)
Supplement: Supplementary file 1 [file Table_1.docx]

**Sarcopenia and stroke outcome: results of a retrospective, cohort study**

*Lea Maria Bumann^1^, Bijan Zendeh Zartoshti^1^, Ulrike Vossmann^1^, Daniel Cantré^2^, Artem Rafaelian^3^, Daniel Dubinski^3^, Alexander Storch^1^, Matthias Wittstock^1^*

**Supplementary Material**

**Supplementary Table S1:** Demographics, baseline clinical characteristics and management data of

study cohort. Sex-specific cutoff points were set at 6.3 mm for male patients and 5.2 mm for female

patients, based on the results of previous published data [19].

**Supplementary Table S2:** Odds ratios for functional outcomes by TMT and by non-sarcopenic vs. sarcopenic patient group.

**Supplementary Table S3:** Univariate statistical analyses of individual risk factors for functional

outcomes at hospital discharge.

**Supplementary Table S24:** Multivariate statistical analyses of risk factors for functional

outcomes at hospital discharge.

**Supplementary Table S5:** Testing of multicollinearity in a multivariate model with mRS 6 as dependent

variable.

**Supplementary Table S6**: Testing of multicollinearity in a ordinal multivariate model with mRS as

dependent variable.

**TABLE S3.** Demographics, baseline clinical characteristics and management data of study cohort. Sex-specific cutoff points were set at 6.3 mm for male patients and 5.2 mm for female patients, based on the results of previous studies^[[1]](#footnote-1)^

|  | **Men** |  |  | **Women** |  |  |
| --- | --- | --- | --- | --- | --- | --- |
|  | **Non-sarcopenic group (n=48)** | **Sarcopenic group (n=32)** | ***P* value** | **Non-sarcopenic group (n=30)** | **Sarcopenic group (n=42)** | ***P* value** |
| **Age (years),** *Median (IQR)* | 68.0 (58.8-77.5) | 76.5 (64.8-83.3) | **0.021^*^** | 68.0 (58.8-79.8) | 82.0 (77.3-86.8) | **<0.001^*^** |
| **BMI (kg/m^2^),** *Median (IQR)* | 26.1 (24.4-29.3) | 26.2 (23.5-29.4) | 0.878* | 27.2 (24.2-34.4) | 24.1 (22.0-29.2) | 0.148**^*^** |
| **TMT (mm),** *mean ± SD* | 7.64 ± 1.04 | 4.94 ± 1.03 | **<0.001*** | 6.36 ± 0.82 | 3.87 ± 0.78 | **<0.001*** |
| **Sarcopenia,** *n (%)*  **CFS at admission***, Median (IQR)* | -  2 (2) | -  2 (2-3) | **-**  **0.008*** | -  2 (2-3) | -  3 (2-4) | **-**  0.101* |
| **Length of hospital stay (days),** *Median (IQR)*  **GCS at admission***, Median (IQR)* | 11.0 (7-15.3)  12.5 (9.3-15) | 11.0 (4.8-17.8)  12.5 (10.3-15) | 0.651^*^  0.533^*^ | 11 (7-14)  10 (6-14) | 10.5 (7-15)  11 (9-15) | 0.819^*^  0.293^*^ |
| **NIHSS at admission,** *Median (IQR)* | 15.5 (9-20.8) | 15.0 (9.5-18) | 0.397^*^ | 18.5 (11-23) | 16 (10.3-19) | 0.177^*^ |
| **Stroke-related parameters** |  |  |  |  |  |  |
| ASPECTS score, *Median (IQR)* | 8 (7-9) | 7 (5-8) | 0.114* | 7.5 (6-8.75) | 8 (7-9) | 0.431* |
| Stroke volume (ml), *Median (IQR)* | 16.45 (4.5-60.7) | 26.8 (11.9-155.8) | 0.056^*^ | 39.0 (7.04-70.5) | 17.7 (1.2-62.9) | 0.193^*^ |
| Thrombectomy, *n (%)* | 18 (37.5%) | 14 (43.8%) | 0.645^$^ | 9 (30.0%) | 15 (35.7%) | 0.800^$^ |
| Systemic thrombolysis+thrombectomy, *n (%)*  mTICI 2b-3, *n (%)* | 30 (62.5%)  41 (85.4%) | 18 (56.3%)  26 (81.3%) | 0.645^$^  0.344^$^ | 21 (70.0%)  26 (86.7%) | 27 (64.3%)  35 (83.3%) | 0.800^$^  0.581^$^ |
| **Complications** |  |  |  |  |  |  |
| Aspiration Pneumonia, *n (%)* | 14 (29.2%) | 16 (50.0%) | 0.098^$^ | 6 (20.0%) | 7 (16.7%) | 0.763^$^ |
| Intracerebral hemorrhage, *n (%)* | 8 (16.7%) | 6 (18.8%) | 1.000^$^ | 2 (6.7%) | 7 (16.7%) | 0.289^$^ |
| **Comorbidities** |  |  |  |  |  |  |
| Arterial hypertension, *n (%)* | 31 (64.6%) | 24 (75.0%) | 0.461**^$^** | 23 (76.7%) | 37 (88.1%) | 0.219^$^ |
| Diabetes mellitus, *n (%)* | 10 (20.8%) | 11 (34.4%) | 0.203^$^ | 7 (23.3%) | 11 (26.2%) | 1.000 ^$^ |
| Hyperlipoproteinemia, *n (%)* | 22 (45.8%) | 14 (43.7%) | 1.000^$^ | 11 (36.7%) | 17 (40.5%) | 0.809^$^ |
| Atrial fibrillation, *n (%)*  *Alcohol abuse, n (%)*  *Smoking, n (%)*  **Outcome**  mRS at discharge, Median (IQR) | 20 (41.6%)  9 (18.8%)  16 (33.3%)  3.0 (2-4.25) | 18 (56,2%)  2 (6.3%)  8 (25.0%)  5.0 (3.75-6) | 0.255**^$^**  0.185^$^  0.466**^$^**  **0.001^$^** | 10 (33.3%)  1 (3.3%)  5 (16.7%)  4.0 (2-5.75) | 26 (61.9%)  0 (0.0%)  4 (9.5%)  4.0 (2.25-6.0) | **0.031^$^**  0,417^$^  0.476^$^  0.710^$^ |

Values are median and interquartile ranges (IQR, in brackets), mean values ± standard deviation (SD) or n (%). Significant findings in bold.

BMI: Body Mass Index, CFS: Clinical Frailty Scale, GCS: Glasgow Coma Scale, NIHSS: National Institute of Health Stroke Scale; mRS: modified ranking scale; mTICI: modified treatment in cerebral infaction, TMT: temporalis muscle thickness.

*Mann-Whintey U-test

$Pearson Chi^2^ or Fisher exact test as appropriate

**TABLE S4.** Odds ratios for functional outcomes by TMT and by non-sarcopenic vs. sarcopenic patient group.

|  | **Outcome rate^a^** | | | | | | |  | **Univariate logistic/ordinal regression^c^** | | | | | | | |  | | **Multivariate logistic/ordinal regression^d^** | | | | | | |
| --- | --- | --- | --- | --- | --- | --- | --- | --- | --- | --- | --- | --- | --- | --- | --- | --- | --- | --- | --- | --- | --- | --- | --- | --- | --- |
|  | **Non-sarcopenic (n=74)** | | **Sarcopenic (n=78)** | | | ***P* value^b^** | |  | **Unadjusted Odds ratio (95% CI)** | | | | | ***P* value** | | |  | | **Adjusted Odds ratio (95% CI)** | | | | ***P* value** | | |
| **TMT (mm) as marker for sarcopenia** |  | | |  | | |  | | |  |  | | | |  | | |  | |  | | | |  | |
| mRS at discharge |  | |  | | |  | |  | 0.81 (0.69-0.95) | | | | | **0.008** | | |  | | 0.79 (0.59-1.04) | | | | 0.094 | | |
| mRS 6 (death) at discharge |  | |  | | |  | |  | 0.74 (0.58-0.92) | | | | | **0.008** | | |  | | 0.70 (0.34-1.31) | | | | 0.280 | | |
| mRS at discharge (men) |  | |  | | |  | |  | 0.70 (0.55-0.89) | | | | | **0.004** | | |  | |  | | | |  | | |
| mRS at discharge (women) |  | |  | | |  | |  | 0.88 (0.67-1.15) | | | | | 0.346 | | |  | |  | | | |  | | |
| **Non-sarcopenic vs. sarcopenic group** | |  | | |  | | |  | | | |  |  | | |  | | | | |  |  | | |  |
| mRS at discharge, *Median (IQR)* | 3.0 (2.0-5.0) | | 5.0 (3.0-6.0) | | | **0.006** | |  | 2.24 (1.26-3.96) | | | | | **0.006** | | |  | | 1.65 (0.66-4.14) | | | | 0.283 | | |
| mRS 6, (death) at discharge *n (%)* | 11 (15.0%) | | 26 (33.0%) | | | **0.008** | |  | 2.86 (1.32-6.56) | | | | | **0.010** | | |  | | 1.59 (0.22-12.03) | | | | 0.641 | | |
| mRS at discharge (men), *Median (IQR)* | 3.0 (2-4.25) | | 5.0 (3.75-6) | | | **0.001** | |  | 4.21 (1.80-9.86) | | | | | **<0.001** | | |  | |  | | | |  | | |
| mRS at discharge (women), *Median (IQR*) | 4.0 (2-5.75) | | 4.0 (2.25-6.0) | | | **0.710** | |  | 1.18 (0.51-2.70) | | | | | 0.702 | | |  | |  | | | |  | | |

mRS: Modified ranking scale. TMT, temporal muscle thickness.

^a^Number and (%) for death rate and median (IQR) for mRS.

^b^*P* values are from Pearson Chi² tests (death rate) or Jonckheere-Terpstra tests (mRS).

^c^Odds ratios and *P* values from univariate ordinal or binary logistic regression analyses. An Odds ratio >1 for the continuous variable (TMT) indicates a higher risk at higher values of TMT, while an Odds ratio >1 for dichotomous risk factor (non-sarcopenic vs. sarcopenic group) indicates a higher risk in the sarcopenic group.

^d^Adjustment of Odds ratios for relevant covariates were performed by multivariate ordinal or binary logistic regression with predictive variables from univariate analyses (covariates for data at hospital discharge: age, NIHSS at admission, Clinical Frailty Score at admission, stroke volume, ASPECT-Score, mTICI, hyperlipoproteinemia, aspiration pneumonia, intracerebral hemorrhage, length of hospital stay; covariates for mRS 6 at discharge: age, NIHSS at admission, Clinical Frailty Score at admission, stroke volume, ASPECT-Score, aspiration pneumonia, length of hospital stay).

**SUPPLEMENTARY TABLE S1.** Univariate statistical analyses of individual risk factors for functional outcomes at hospital discharge.

|  | **mRS^a^** | |  | **mRS 0-3^b^** | |  | | **mRS 4-6^b^** | | |  | | **mRS 6 (in-hospital death)^b^** | | |  |
| --- | --- | --- | --- | --- | --- | --- | --- | --- | --- | --- | --- | --- | --- | --- | --- | --- |
|  | **Common odds ratio (95% CI)** | ***P* value** |  | **Odds ratio (95% CI)** | ***P* value** | |  | | **Odds ratio (95% CI)** | ***P* value** | |  | | **Odds ratio (95% CI)** | ***P* value** | |
| **Male/female** | 1.14 (0.65-2.01) | 0.643 |  | 1.02 (0.54-1.95) | 0.945 | |  | | 1.03(0.54-1.96) | 0.931 | |  | | 1.43 (0.68-3.02) | 0.350 | |
| **Age (years)** | 1.04 (1.02-1.06) | **<0.001** |  | 0.98 (0.96-1.00) | 0.071 | |  | | 1.02 (0.99-1.04) | 0.077 | |  | | 1.05 (1.02-1.09) | **0.003** | |
| **BMI** | 1.06 (0.99-1.12) | 0.058 |  | 0.95 (0.88-1.01) | 0.121 | |  | | 1.05 (0.98-1.13) | 0.167 | |  | | 1.04 (0.97-1.12) | 0.232 | |
| **TMT (mm)** | 0.81 (0.69-0.95) | **0.008** |  | 1.16 (0.96-1.39) | 0.125 | |  | | 0.87 (0.72-1.04) | 0.126 | |  | | 0.74 (0.58-0.92) | **0.008** | |
| **Sarcopenia** | 2.24 (1.26-3.96) | **0.006** |  | 0.50 (0.26-0.96) | **0.038** | |  | | 1.89 (0.99-3.64) | 0.056 | |  | | 2.86 (1.32-6.56) | **0.010** | |
| **CFS at admission** | 1.77 (1.29-2.57) | **0.001** |  | 0.57 (0.40-0.78) | **0.001** | |  | | 1.77 (1.29-2.57) | **0.001** | |  | | 1.31 (0.99-1.74) | **0.047** | |
| **NIHSS at admission** | 1.13 (1.08-1.19) | **<0.001** |  | 0.88 (0.83-0.93) | **<0.001** | |  | | 1.14 (1.08-1.20) | **<0.001** | |  | | 1.12 (1.05-1.19) | **<0.001** | |
| **GCS at admission** | 0.86 (0.78-0.93) | **<0.001** |  | 1.16 (1.05-1.30) | **0.005** | |  | | 0.86 (0.77-0.94) | **0.003** | |  | | 0.87 (0.78-0.96) | **0.006** | |
| **Stroke volume (ml)** | 1.02 (1.02-1.03) | **<0.001** |  | 0.98 (0.97-0.99) | **<0.001** | |  | | 1.02 (1.02-1.04) | **<0.001** | |  | | 1.02 (1.01-1.03) | **<0.001** | |
| **ASPECT-score** | 0.63 (0.52-0.75) | **<0.001** |  | 1.55 (1.24-2.03) | **<0.001** | |  | | 0.64 (0.49-0.80) | **<0.001** | |  | | 0.65 (0.52-0.78) | **<0.001** | |
| **Thrombektomy** | 0.91 (0.51-1.61) | 0.740 |  | 1.41 (0.72-2.79) | 0.317 | |  | | 0.68 (0.34-1.33) | 0.262 | |  | | 1.10 (0.51-2.44) | 0.805 | |
| **Systemic thrombolysis+thrombectomy** | 0.91 (0.51-1.61) | 0.740 |  | 1.41 (0.72-2.79) | 0.317 | |  | | 0.68 (0.34-1.33) | 0.262 | |  | | 1.10 (0.51-2.44) | 0.805 | |
| **mTICI 2b-3** | 0.37 (0.17-0.75) | **0.010** |  | 4.55 (1.62-16.31) | **0.009** | |  | | 0.21 (0.06-0.60) | **0.007** | |  | | 0.47 (0.19-1.21) | 0.107 | |
| **Aspiration pneumonia** | 4.65 (2.45-9.01) | **<0.001** |  | 0.08 (0.02-0.22) | **<0.001** | |  | | 9.66 (3.82-29.72) | **<0.001** | |  | | 2.91 (1.33-6.38) | **0.007** | |
| **Intracerebral hemorrhage** | 2.37 (1.10-5.28) | **0.031** |  | 0.32 (0.10-0.86) | **0.033** | |  | | 2.46 (0.96-7.19) | 0.075 | |  | | 1.84 (0.68-4.69) | 0.210 | |
| **Atrial hypertension** | 1.65 (0.85-3.22) | 0.143 |  | 0.73 (0.35-1.54) | 0.406 | |  | | 1.32 (0.63-2.79) | 0.461 | |  | | 1.51 (0.63-4.07) | 0.379 | |
| **Diabetes mellitus** | 1.69 (0.88-3.27) | 0.115 |  | 0.68 (0.31-1.43) | 0.316 | |  | | 1.32 (0.63-2.81) | 0.469 | |  | | 1.85 (0.82-4.12) | 0.132 | |
| **Hyperlipoproteinemia** | 0.59 (0.33-1.03) | 0.064 |  | 1.31 (0.68-2.52) | 0.416 | |  | | 0.80 (0.42-1.53) | 0.501 | |  | | 0.25 (0.09-0.58) | **0.002** | |
| **Atrial fibrillation** | 0.99 (0.57-1.73) | 0.970 |  | 0.93 (0.49-1.78) | 0.945 | |  | | 1.01 (0.53-1.93) | 0.966 | |  | | 0.75 (0.35-1.57) | 0.447 | |
| **Alcohol abuse** | 0.74 (0.28-1.95) | 0.534 |  | 1.37 (0.41-4.59) | 0.599 | |  | | 0.75 (0.22-2.51) | 0.633 | |  | | 0.26 (0.01-1.42) | 0.208 | |
| **Smoking** | 0.88 (0.46-1.69) | 0.704 |  | 0.98 (0.44-2.14) | 0.965 | |  | | 1.05 (0.49-2.33) | 0.896 | |  | | 0.36 (0.10-1.00) | **0.043** | |
| **Length of hospital stay** | 0.96 (0.93-0.99) | **0.020** |  | 1.02 (0.99-1.07) | 0.230 | |  | | 0.97 (0.93-1.01) | 0.128 | |  | | 0.74 (0.66-0.83) | **<0.001** | |

BMI: body mass index; CFS: Clinical Frailty Scale, GCS: Glasgow Coma Scale, mRS: modified ranking scale; mTICI: modified treatment in cerebral infarction, NIHSS: National Institute of Health Stroke Scale; TMT: temporalis muscle thickness.

^a^Odds ratios and *P* values from univariate logistic ordinal regression analyses. An Odds ratio >1 for sex as a risk factor indicates a higher risk for the outcome/death in women, an Odds ratio >1 for dichotomous risk factors indicates a higher risk in the presence of the respective risk factor, while an Odds ratio >1 for continuous variables indicates a higher risk at higher values of the potential risk factor.

^b^Odds ratios and *P* values from univariate logistic binary regression analyses. An Odds ratio >1 for sex as a risk factor indicates a higher risk for the outcome/death in women, an Odds ratio >1 for dichotomous risk factors indicates a higher risk in the presence of the respective risk factor, while an Odds ratio >1 for continuous variables indicates a higher risk at higher values of the potential risk factor.

**SUPPLEMENTARY TABLE S2.** Multivariate statistical analyses of risk factors for functional outcomes at hospital discharge.

|  | **mRS^a^** | |  | **mRS 6 (in-hospital death)^b^** | |
| --- | --- | --- | --- | --- | --- |
|  | **Common odds ratio (95% CI)** | ***P* value** |  | **Odds ratio (95% CI)** | ***P* value** |
| **Age (years)** | 1.02 (0.98-1.06) | 0.335 |  | 0.98 (0.90-1.06) | 0.528 |
| **TMT (mm)** | 0.79 (0.59-1.04) | 0.094 |  | 0.70 (0.34-1.31) | 0.280 |
| **NIHSS at admission** | 1.06 (0.97-1.04) | 0.195 |  | 1.02 (0.83-1.28) | 0.875 |
| **GCS at admission** | 0.99 (0.86-1.13) | 0.833 |  | 0.88 (0.62-1.26) | 0.446 |
| **CFS at admission** | 1.48 (1.01-2.16) | **0.046** |  | 1.39 (0.67-2.85) | 0.362 |
| **Stroke volume** | 1.02 (1.01-1.03) | **0.003** |  | 1.01 (0.99-1.04) | 0.142 |
| **ASPECT-Score** | 0.82 (0.62-1.09) | 0.174 |  | 0.70 (0.41-1.11) | 0.146 |
| **mTICI 2b-3** | 0.27 (0.05-1.39) | 0.117 |  | - | - |
| **Hyperlipoproteinemia** | - | - |  | 0.98 (0.17-5.93) | 0.978 |
| **Aspiration pneumonia** | 6.69 (2.09-21.55) | **0.001** |  | 7.06 (0.86-93.85) | 0.088 |
| **Intracerebral hemorrhage** | 2.43 (0.81-7.25) | 0.112 |  | - | - |
| **Length of hospital stay (days)** | 0.95 (0.90-1.00) | 0.054 |  | 0.73 (0.56-0.87) | **0.003** |
| **Smoking** | - | - |  | 0.27 (0.02-3.04) | 0.318 |

CFS: Clinical Frailty Scale, GCS: Glasgow Coma Scale, NIHSS: National Institute of Health Stroke Scale; mRS: modified ranking scale; mTICI: modified treatment in cerebral infarction, TMT: temporalis muscle thickness.

^a^Odds ratio >1 for dichotomous risk factors indicates a higher risk in the presence of the respective risk factor, while an Odds ratio >1 for continuous variables indicates a higher risk at higher values of the potential risk factor.

^b^Odds ratios and *P* values from multivariate binary logistic regression analyses using the variables resulted statistically significant in the univariate comparison as predictors. An Odds ratio >1 for dichotomous risk factors indicates a higher risk in the presence of the respective risk factor, while an Odds ratio >1 for continuous variables indicates a higher risk at higher values of the potential risk factor

**Table S5**: Testing of multicollinearity in a multivariate model with mRS 6 as dependent variable.

| mRS 6 (TMT as marker for sarcopenia) | | mRS 6 (sarcopenic/non-sarcopenic) | |
| --- | --- | --- | --- |
|  | **VIF** |  | **VIF** |
| Age | 1.69 | Age | 1.61 |
| TMT | 1.93 | Sarcopenia | 1.55 |
| NIHSS at admission | 3.40 | NIHSS at admission | 3.63 |
| GCS at admission | 3.44 | GCS at admisssion | 3.59 |
| CFS at admission | 1.76 | CFS at admission | 1.60 |
| Stroke volume | 1.48 | Stroke volume | 1.46 |
| ASPECT-score | 1.33 | ASPECT-score | 1.43 |
| Hyperlipoproteinemia | 1.13 | Hyperlipoproteinemia | 1.13 |
| Aspiration pneumonia | 1.70 | Aspiration pneumonia | 1.85 |
| Length of hospital stay | 1.57 | Length of hospital stay | 1.68 |
| Smoking | 1.55 | Smoking | 1.66 |

mRS: Modified ranking scale, VIF: Variance inflation factor, CFS: Clinical Frailty Scale, GCS: Glasgow Coma Scale,

NIHSS: National Institute of Health Stroke Scale; ASPECT-score: Alberta; mTICI: modified treatment in cerebral

infarction, TMT: temporalis muscle thickness.

**Tabelle S6**: Testing of multicollinearity in a ordinal multivariate model with mRS as dependent variable.

| mRS (TMT as marker for sarcopenia) | | mRS (sarcopenic/non-sarcopenic) | |
| --- | --- | --- | --- |
|  | **VIF** |  | **VIF** |
| Age | 1.47 | Age | 1.40 |
| TMT | 1.38 | Sarcopenia | 1.25 |
| NIHSS at admission | 2.19 | NIHSS at admission | 2.14 |
| GCS at admission | 2.18 | GCS at admission | 2.10 |
| CFS at admission | 1.12 | CFS at admission | 1.11 |
| Stroke volume | 1.27 | Stroke volume | 1.23 |
| ASPECT-score | 1.21 | ASPECT-score | 1.22 |
| mTICI 2b-3 | 1.43 | mTICI 2b-3 | 1.42 |
| Aspiration pneumonia | 1.38 | Aspiration pneumonia | 1.35 |
| Intracerebral hemorrhage | 1.17 | Intracerebral hemorrhage | 1.17 |
| Length of hospital stay | 1.30 | Length of hospital stay | 1.29 |

mRS: Modified ranking scale, VIF: Variance inflation factor, CFS: Clinical Frailty Scale, GCS: Glasgow Coma Scale, NIHSS: National Institute of Health Stroke Scale; ASPECT-score: Alberta; mTICI: modified treatment in cerebral infarction, TMT: temporalis muscle thickness.

1. [↑](#footnote-ref-1)
